# Safety Evaluation of Hemoglobin-Albumin Cluster “HemoAct” as a Red Blood Cell Substitute

**DOI:** 10.1038/srep12778

**Published:** 2015-07-29

**Authors:** Risa Haruki, Takuya Kimura, Hitomi Iwasaki, Kana Yamada, Ikuo Kamiyama, Mitsutomo Kohno, Kazuaki Taguchi, Saori Nagao, Toru Maruyama, Masaki Otagiri, Teruyuki Komatsu

**Affiliations:** 1 Department of Applied Chemistry, Faculty of Science and Engineering, Chuo University, 1-13-27 Kasuga, Bunkyo-ku, Tokyo 112-8551, Japan; 2 Department of Thoracic Surgery, School of Medicine, Keio University, 35 Shinanomachi, Shinjuku-ku, Tokyo 160-8582, Japan; 3 Faculty of Pharmaceutical Sciences, Sojo University, 4-22-1 Ikeda, Nishi-ku, Kumamoto 860-0082, Japan; 4 Department of Biopharmaceutics, Graduate School of Pharmaceutical Sciences, Kumamoto University, 5-1 Oe-Honmachi, Chuo-ku, Kumamoto 862-0973, Japan

## Abstract

A hemoglobin (Hb) wrapped covalently by human serum albumins (HSAs), a core–shell structured hemoglobin-albumin cluster designated as “HemoAct”, is an O_2_-carrier designed for use as a red blood cell (RBC) substitute. This report describes the blood compatibility, hemodynamic response, and pharmacokinetic properties of HemoAct, and then explains its preclinical safety. Viscosity and blood cell counting measurements revealed that HemoAct has good compatibility with whole blood. Intravenous administration of HemoAct into anesthetized rats elicited no unfavorable increase in systemic blood pressure by vasoconstriction. The half-life of ^125^I-labeled HemoAct in circulating blood is markedly longer than that of HSA. Serum biochemical tests conducted 7 days after HemoAct infusion yielded equivalent values to those observed in the control group with HSA. Histopathologic inspections of the vital organs revealed no marked abnormality in their tissues. All results indicate that HemoAct has sufficient preclinical safety as an alternative material for RBC transfusion.

Japan is prone to natural disasters. When a great earthquake occurs, large amounts of blood are required immediately. Nevertheless, the donated blood cannot be stored for long periods. For example, the preservation limit of red blood cells (RBC) has been established as 21 days at 2–6 °C^1^. Therefore, sufficient blood might not be available in the event of a widespread disaster. Another problem is that Japan’s declining birthrate and aging society make it difficult to maintain a stable blood transfusion system. Currently, 85% of blood products in Japan are used for patients aged fifty years old or older^2^. The number of elderly people will continue to increase, although the population of younger blood donors is expected to decrease. The Japanese Red Cross Society predicts a blood shortage equivalent to 890,000 people per year in 2027^3^. Consequently, a blood substitute, in particular an RBC substitute, is needed (i) which can be stored for long period, (ii) which presents no risk of virus infection, and (iii) which is useful for anyone irrespective of blood type. Such an artificial O_2_-carrier is required as a primary measure for crisis management, and is required as a medical measure to supplement blood transfusion treatment.

Since the 1980 s, hemoglobin (Hb)-based O_2_-carriers (HBOCs) of several kinds have been manufactured and evaluated^4^,^5^,^6^, such as crosslinked Hb^7^,^8^, polymerized Hb^9^,^10^,^11^, and poly(ethyleneglycol)-conjugated Hb (PEG-Hb)^12^,^13^,^14^. Clinical studies of some products reached Phase-III, but side-effects (pressor response) and low efficacy have prevented their practical application^5^,^10^,^15^,^16^. The increase in systemic blood pressure observed after the infusion is probably caused in part by vasoconstriction induced by Hb diffusion into the extravascular space and scavenging endothelial-derived relaxing factor, nitric oxide (NO)^17^,^18^.

Recently, we synthesized a covalent core–shell structured protein cluster comprising Hb in the center and human serum albumin (HSA) at the periphery as a unique HBOC (Fig. 1)^19^,^20^,^21^. The average HSA/Hb ratio of one cluster was 3.0 ± 0.2 (Hb-HSA_*3*_). The O_2_-binding property was well defined. We designated this hemoglobin–albumin cluster as “HemoAct”. Actually, HSA is the most prominent plasma protein in the bloodstream (approximately 4–5 g/dL), playing the role of maintaining colloid osmotic pressure, as well as transporting various metabolites and drugs^22^,^23^. Because HSA contains only one sulfhydryl group of Cys at position 34, we exploited a heterobifunctional crosslinker, *N*-succinimidyl-4-(*N*-maleimidomethyl)cyclohexane-1-carboxylate (SMCC), as a connector between the Cys-34 residue of HSA and the surface Lys amino groups of Hb^19^,^20^. The formulated HemoAct has satisfactorily negative surface net charge (*p*I: 5.1). Therefore it might not be leaked from the vasculature walls because of the electrostatic repulsion against the glomerular basement membrane around the endothelial cells. Probably, intravenous transfusion of HemoAct would not elicit the acute increase of the blood pressure and would support a long period of blood circulation. To evaluate the preclinical safety of the HemoAct solution as an RBC substitute, we examined the blood compatibility, hemodynamic response, and pharmacokinetic property of this new O_2_-carrier.

## Results and Discussion

### Blood compatibility

**Figure 2:**
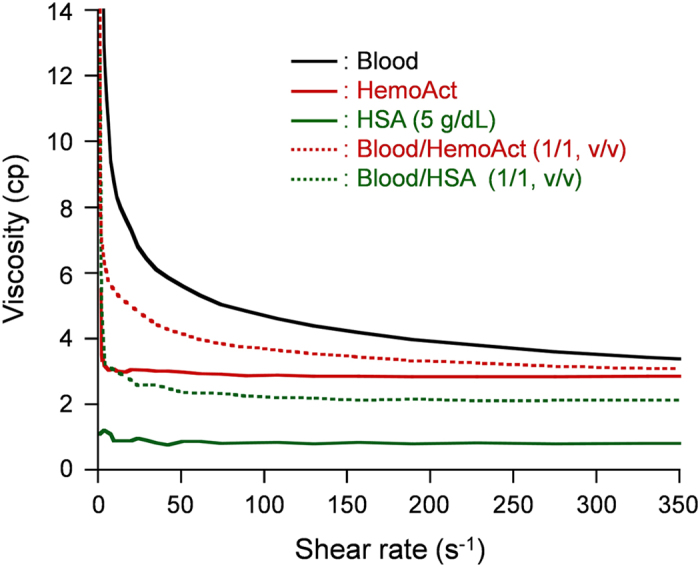
Viscosity of the HemoAct solution. Correlations between the shear rate and viscosity of the HemoAct solution and blood/HemoAct mixture suspension at 37 °C.

Many clinical disorders alter blood viscosity, thereby causing RBC aggregation^24^. In fact, plasma proteins are important blood components affecting blood viscosity. The viscosity of the HemoAct solution (20 g/dL, [Hb] = 5.0 g/dL) is dependent on the shear rate, indicating that this O_2_-carrier is a Newtonian fluid, just as HSA is (5 g/dL) (Fig. 2). The viscosity at 230 s^-1^, the shear rate in the human arterial wall, was ascertained as 2.8 cP, which is lower than that of blood (3.8 cP). The HemoAct solution was mixed with freshly drawn whole blood of rats (1/1, v/v). A homogeneous blood/HemoAct suspension exhibited non-Newtonian viscosity, which obeyed a nonlinear correlation to the shear rate. The viscosity was reasonably high: 3.3 cP at 230 s^-1^. No precipitation was observed for 6 h at 37 °C. These results indicate that HemoAct has good compatibility with whole blood.

**Figure 3:**
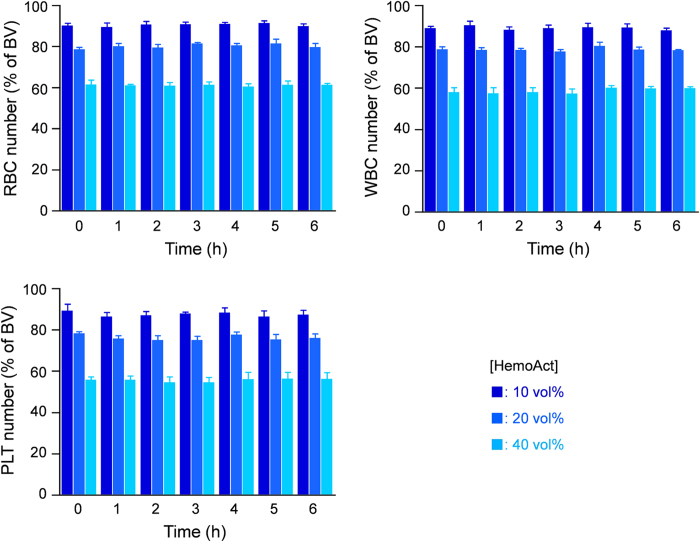
Blood compatibility. Time course of blood cell numbers in blood/HemoAct mixture suspension ([HemoAct] = 10, 20, and 40 vol%) at 37 °C. The results are shown as percentages of (cell number with HemoAct)/(cell number without HemoAct (BV at each time-point)). Each bar represents the mean ± SD (*n* = 3). Basal values at 0 h are 828 ± 25 cells/μL in RBC group, 38 ± 11 × 10^2^ cells/μL in WBC group, and 97 ± 11 × 10^4^ cells/μL in the PLT group.

The rapid increase and decrease of the blood cell number causes blood disorder. We measured the number of blood cell components [RBC, white blood cell (WBC), and platelet (PLT)] of the blood/HemoAct mixture suspensions (9/1, 8/2, and 6/4, v/v) *in vitro*. The numbers of RBC, WBC, and PLT decreased in proportion to their respective dilution ratios: approximately 90, 80, and 60% of the baseline value (BV) (Fig. 3). The percentage of (cell number with HemoAct)/(cell number without HemoAct (BV at each time-point)) remained constant for 6 h at 37 °C. The results were identical to those observed in control experiments with HSA (not shown).

The influence of HemoAct on blood coagulation was also evaluated using measurements of the prothrombin time (PT) and activated partial thromboplastic time (APTT). The PT tests assess the extrinsic and common pathways of the coagulation cascade. The APTT tests evaluate the intrinsic and common pathways. Using both tests, the integrated function of all the blood coagulation factors can be examined^25^. Mixing of HemoAct with the whole blood (1/1, v/v) did not change the PT and APTT (Fig. S1). The values are almost identical to those of the control group with HSA. To standardize the results, the international normalized ratio (INR), which is (PT with sample)/(PT without sample) raised to the power of the International Sensitivity Index (ISI), was exploited^26^. The INR value higher than 1.5 suggests the occurrence of hemorrhage or disease. In our experiments, all INR values were 0.98–1.13. Based on these findings, we conclude that HemoAct shows no unfavorable interaction to the blood cell components, and that it does not obstruct the blood coagulation function.

### Hemodynamic response

**Figure 4:**
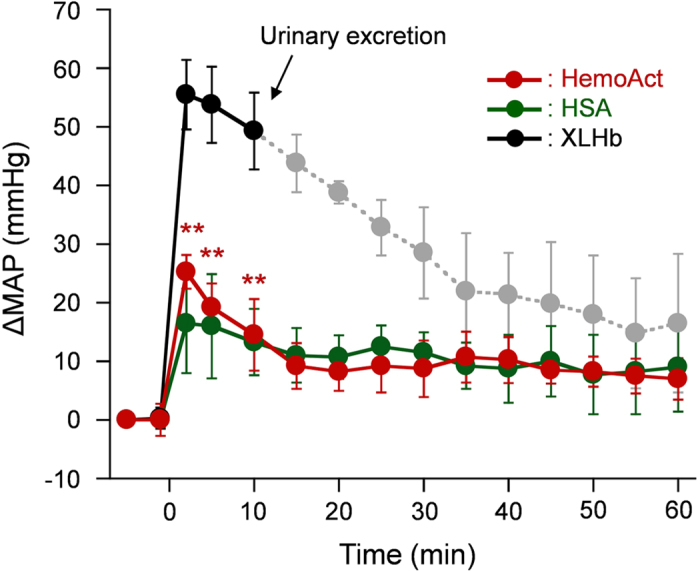
Hemodynamic response. Difference of mean arterial pressure (ΔMAP) from the basal value after intravenous administration of HemoAct, HSA, and XLHb solutions to rats. Each data point represents the mean ± SD (*n* = 4). **p < 0.01 vs. XLHb. Basal values are 84.5 ± 4.4 mmHg in the HemoAct group, 85.0 ± 7.3 mmHg in the HSA group, and 87.5 ± 5.1 mmHg in the XLHb group. From 10 min after the infusion, Hb excretion was observed in the XLHb group. Therefore (i) the data points are shown in light grey; (ii) no precise comparison between HemoAct group and XLHb group was performed.

To avoid the depletion of NO by the extravasation of Hbs, surface-modified Hbs with PEG (PEG-Hbs) have been designed and synthesized^12^,^13^,^14^. By virtue of their greater molecular size, their extravasation is reasonably attenuated^12^. Nonetheless, the high viscosity of PEG-Hb can substantially influence the plasma volume and shear stress on the capillary wall, which can affect blood pressure. We injected the HemoAct solution (20 g/dL) into anesthetized rats (6 ml/kg) and monitored their mean arterial pressure (MAP) for 60 min. As expected, only a transient alternation in MAP was observed after administration of HemoAct (Fig. 4). The 25.3 ± 2.9 mmHg elevation of ∆MAP from the basal value was followed by a decrease to 10 mmHg within 15 min. It remained constant during the monitoring period. The response is precisely the same result as that observed after infusion of HSA (20 g/dL). In contrast, the administration of ββ-crosslinked Hb (XLHb, 5 g/dL) is associated with (i) an immediate and remarkable increase in ∆MAP (55.5 ± 5.9 mmHg) and (ii) urinary excretion of Hb beginning 10 min after the injection.

This non-vasopressor effect of HemoAct, which is remarkable compared to that of XLHb, appears to be attributable to the negative surface net charge and increased molecular mass of the cluster. HSA shows low vascular permeability of less than 1/100 that for Hb ascribed to the electrostatic repulsion between the albumin surface and the glomerular basement membrane around the endothelial cells^27^. The isoelectric point of HemoAct (*p*I = 5.1) is comparable to that of HSA. Moreover, the molecular mass (26.4 kDa) of HemoAct is four times larger than that of HSA (66.5 kDa)^19^. Consequently, the leakage of negatively charged and huge HemoAct into the extravascular space must be suppressed. In contrast, the small XLHb having neutral surface net charge passes through the vascular endothelium and contributes considerably to the consumption of NO, thereby increasing the blood pressure. They also pass through the renal glomerulus soon after the infusion, thereby inducing excretion of the observable quantities of Hb in urine.

Other mechanisms for the rise in MAP by the Hb products are presumed to be (i) the autoregulatory response to high arteriole O_2_-level^28^,^29^,^30^ and (ii) the decrease in NO production by diminished shear stress on the vasculature wall^31^. Regarding the first hypothesis, Winslow *et al.* reported that HBOCs with a low O_2_-affinity engender excessive O_2_ release in the arterioles and produce autoregulatory vasoconstriction^29^. The O_2_-affinity of HemoAct is high enough (*P*_50_: 9 Torr) that excessive O_2_ offloading in the arterioles is unlikely. In the second hypothesis, the prompt flow by the administration of HemoAct with a low viscosity (compared to blood) might decrease the shear stress on the vasculature wall. However, the total blood volume increased less than 11% in these top-load experiments. Therefore the change of viscosity was regarded as negligible.

### Pharmacokinetic properties

**Figure 5:**
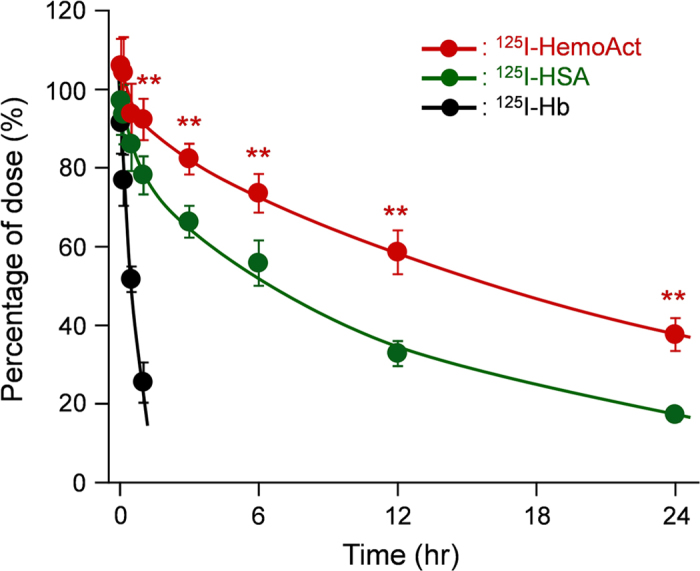
Blood retention. Relative plasma concentration of ^125^I-HemoAct, ^125^I-HSA, and ^125^I-Hb after intravenous administration to rats. Each data point represents the mean ± SD (*n* = 6). **p < 0.01 vs. ^125^I-HSA.

The ^125^I-labeled HemoAct was injected into rats to assess blood retention and tissue distribution. The ^125^I-labeled Hb, as a control material, was cleared rapidly from circulation (Fig. 5). The half-life (*T*_1/2_) was only 0.53 h, which was comparable to the value reported previously by Pang *et al.*^32^ The time course of the plasma concentration of HemoAct showed very slow kinetics in two phases. The pharmacokinetic parameters were determined using a non-compartment model (Table 1). The *T*_1/2_ of HemoAct was markedly long (18.5 h) and 1.7-fold greater than that of HSA (*T*_1/2_ = 11.0 h). As described earlier, the negative surface net charge and large molecular size of HemoAct prevent not only extravasation through the vascular endothelium, but also filtration by the renal glomerulus. Accompanied by the decrease of CL and *V*_dss_ values, the AUC and *T*_1/2_ of HemoAct were increased relative to those of HSA. We are convinced that the superior blood retention property of HemoAct is attributable to suppression of movement to the extravascular space and renal filtration.

**Figure 6:**
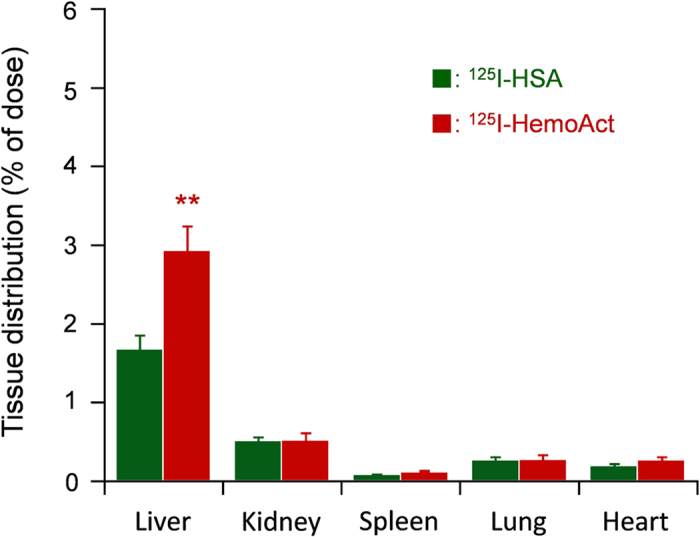
Tissue distribution. Tissue (vital organs) distribution of radioactivity (% of dose) at 24 h after intravenous administration of ^125^I-HemoAct to rats. Each bar shows the mean ± SD (*n* = 6). **p < 0.01 vs. ^125^I-HSA.

Figure 6 depicts the tissue (vital organs) distribution of HemoAct at 24 h after administration. HemoAct and HSA were similarly distributed in the major organs. Careful assessments revealed that the extent of HemoAct accumulation in the liver was greater than that of HSA. Rennen *et al.* reported that the large proteins are cleared with predominant uptake by the liver^33^. We inferred that higher hepatic distribution of HemoAct is attributable to the large molecular volume of the cluster. This result is consistent with our previously reported pharmacokinetic study of the HSA dimer^34^,^35^.

### Serum biochemical tests and histopathologic observations

The serum biochemical test is an inspection to measure the abnormality of a physical condition and organ. All animals given the HemoAct solution (20 g/dL, 6 mL/kg) were alive for 7 days after the infusion. During the measurement period, no remarkable change was found in their appearance or behavior. Although anesthesia and surgical operation temporarily decreased the body weight of rats after 1 day (279 ± 14 g→265 ± 13 g), the body weight increased gradually thereafter and reached 318 ± 14 g after a week (Fig. S2). The growth processes were almost identical to those observed in the control groups with HSA and without infusion (sham-operation).

All 26 analytes of the serum biochemical tests after 7 days from the administration exhibited almost identical data to those of the control groups (Table S1). The weights of major organs (liver, kidney, spleen, lungs, and heart) recovered from the rats were also nearly equal to the values of the control groups (Fig. S3).

Furthermore, microscopic observations of the stained specimens of these vital organs demonstrated no histopathologic disorder in their tissues (Fig. S4). The HemoAct molecule disappeared within a week. Some abnormality might be detected in the liver if the excess hemes of HemoAct were not decomposed by hemeoxygenase. Nevertheless, we were unable to find even a small difference between the HemoAct and HSA groups. This similarity is supported by the values of liver function markers (AST, ALT, and γ-GTP) revealed by serum biochemical tests. Although more research must be done to elucidate the metabolism, the present results imply that the administration of HemoAct induces no negative side effects or middle-term toxic reactions in vital organs.

## Conclusions

The blood/HemoAct mixture suspension (1/1, v/v) was a non-Newtonian fluid similar to whole blood, exhibiting viscosity of 3.3 cP (at a shear rate of 230 s^−1^). The addition of 40 vol% HemoAct into the blood did not affect the quantities of RBC, WBC, and PLT for 6 h at 37 °C. The coagulation function of the blood was also maintained. The HemoAct solution shows good compatibility with blood *in vitro*. The administration of HemoAct to anesthetized rats caused a slight change in MAP, which is identical to that observed in the control group with HSA. This hemodynamic response contrasts against the fact that drastic hypertension and urinary excretion of Hb occurred after infusion of XLHb. The *T*_1/2_ of HemoAct was 1.7-fold longer than that of HSA. The non-vasopressor response and superior blood retention property of HemoAct are attributed to the negative surface net charge and larger molecular size of the cluster. The serum biochemical parameters closely resembled those of the control groups with HSA and without infusion. Histopathologic inspections proved that HemoAct produced no negative side effects in any major organ. These results underscore the preclinical safety of HemoAct, and enable us to undertake further advanced *in vivo* study of this O_2_-carrying protein cluster as a blood replacement.

## Methods

### Hb-HSA_3_ solution

The phosphate-buffered saline solution of HemoAct (20 g/dL, pH 7.4, HSA/Hb = 3.0 ± 0.2 (mol/mol)) was prepared according to our previously reported procedures using purified bovine Hb and human serum albumin (HSA, Japan Blood Products Organization)^19^,^20^. The procedures used for synthesis of ββ-crosslinked Hb (XLHb) were described in Supplementary Information. The O_2_ affinity (*P*_50_: O_2_-partial pressure where Hb is half-saturated with O_2_) and Hill coefficient (*n*) were 9.0 Torr and 1.5, respectively, as determined using an automatic recording system for blood O_2_-equilibrium curves (Hemox-Analyzer; TCS Scientific Corp.) using PBS (pH 7.4) at 37 °C.

### Animal experiments

Animal studies were reviewed and approved by the Animal Care and Use Committee of Keio University, Sojo University, or Kumamoto University. The care and handling of the animals were done in accordance with NIH guidelines.

### Viscosity measurements

Correlation between the shear rate and viscosity of the HemoAct solution (20 g/dL) was measured using a rheometer system (Physica MCR101; Anton Paar Japan K.K.) at 37 °C. Fresh whole blood was obtained from Wistar rats (ca. 275 g, male; Charles River Laboratories Japan, Inc.) and was stored in EDTA-2 K coated blood collection tubes. The HemoAct solution was added to the whole blood (1/1, v/v). Then the viscosity of the blood/HemoAct mixture suspension (0.4 mL) was measured (measuring points, 36; shear rate, 0–700 s^−1^; measuring interval, 25 s). As control groups, the HSA (5 g/dL) solution, blood, and blood/HSA (1/1, v/v) suspensions were measured under the same conditions.

### Blood cell numbers

Fresh whole blood was obtained from Wistar rats (ca. 275 g, male) and stored in EDTA-2 K coated blood collection tubes. The HemoAct solution was added to the whole blood at 0, 10, 20, and 40 vol% concentrations (total volume 1.5 mL each). Each individual sample was incubated at 37 °C in an incubator. At the time-points of 0, 1, 2, 3, 4, 5, and 6 h after the mixing, 0.1 mL of the sample was drawn from each group ([HemoAct] = 0, 10, 20, and 40 vol%), and the quantities of blood cell components (RBC, WBC, and PLT) were assessed using an automated hematology analyzer for animals (pocH-100*iV Diff*; Sysmex Corp.) (*n* = 3). As control groups, the blood suspensions with HSA (5 g/dL) ([HSA] = 0, 10, 20, and 40 vol%) were also measured (*n* = 3). The results are shown as a percentage of (cell number with sample)/(cell number without sample (basal value at each time-point)).

### Prothrombin time (PT) and activated partial thromboplastin time (APTT)

Fresh whole blood was obtained from Wistar rats (ca. 275 g, male) and was stored in sodium citrate coated blood collection tubes. The HemoAct solution was added to the whole blood at 0, 10, 20, and 40 vol% concentration (total volume 1.2 mL each) in different blood collection tubes (B-11; BML Inc.). These samples were centrifuged (2,800 rpm, 15 min). Then the supernatant (0.6 mL) was transferred to blood collection tube (S-1; BML Inc.) and was frozen at −80 °C. As control groups, blood suspensions with HSA (5 g/dL) ([HSA] = 0, 10, 20, and 40 vol%) were also prepared. The PT and APTT measurements were performed by BML Inc. (Tokyo) (*n* = 4 each).

### Blood pressure response

Wistar rats (264 ± 3 g, male) were placed on a heating pad under an inhalation anesthesia with 1.0% sevoflurane. After an incision was made in the neck, a heparinized catheter (SP31 tubing, OD 0.8 mm, ID 0.2 mm) was introduced into the right common carotid artery and was connected to a blood pressure measurement system (KN-213/KN-212; Natsume Seisakusho Co., Ltd., Tokyo) for continuous recording of the mean arterial pressure (MAP). Another catheter (SP-31) was inserted into the right jugular vein for sample injection.

After stabilization of the animal condition under 0.5% sevoflurane, the HemoAct solution (20 g/dL) was administered intravenously via the jugular vein (6 mL/kg, 1 mL/min, *n* = 4). During the injection, no remarkable acute reaction was observed in the appearance of the animal. The MAP was monitored at the time-points of 5 min, 1 min before the infusion, and 2 min, and 5–60 min (5 min intervals) after the infusion. As control groups, the ββ-crosslinked Hb (XLHb) solution (5 g/dL) and HSA (20 g/dL) were also applied to similarly treated rats [XLHb group (264 ± 13 g, 6 mL/kg, 1 mL/min, *n* = 4) and HSA group (258 ± 6 g, 6 mL/kg, 1 mL/min, *n* = 4)].

### Serum biochemical tests and histopathologic observations

Wistar rats (279 ± 14 g, male) were anesthetized with an intraperitoneal injection of sodium pentobarbital. The HemoAct solution (20 g/dL) was injected intravenously via a tail vein (6 mL/kg, 1 mL/min, *n* = 3). Then the appearances and weights of the animals were observed for 7 days. During the measurement period, the animals were housed in cages and provided with continuous access to food and water in an air-conditioned room on a 12 h dark/light cycle. After 7 days, the rats were anesthetized again with an intraperitoneal injection of sodium pentobarbital. A heparinized catheter (SP31 tubing, OD 0.8 mm, ID 0.2 mm) was introduced into the abdominal aorta. Then blood was withdrawn using a nontreated syringe. The blood was transferred to a vacuum blood collection tube (B-2; BML Inc.) and was centrifuged (2,800 rpm, 15 min, 4 °C) to remove the blood cell components. The obtained serum was stored in the tube (S-1; BML Inc.) at 4 °C. The samples were subjected to a total of 26 blood biochemical assays by BML Inc. (Tokyo): total protein, albumin, albumin/globulin ratio, aspartate aminotransferase (AST), alanine aminotransferase (ALT), γ-glutamyltransferase (γ-GTP), total bilirubin, direct bilirubin, creatinine, urea nitrogen, uric acid, amylase, total cholesterol, free cholesterol, β-lipoprotein, high-density lipoprotein (HDL)-cholesterol, triglyceride, total lipid, free fatty acid, phospholipid, K, Ca, inorganic P, Mg, Fe, and Cu. The vital organs (liver, kidney, spleen, lung, and heart) were isolated and weighed. As a control group, the 20 g/dL HSA solution was administered similarly into rats (269 ± 17 g, 6 mL/kg, 1 mL/min, *n* = 3). Three rats (273 ± 14 g) without infusion (operation only) were established as a sham-operated group.

In addition, paraffin section specimens were prepared from 10% formalin-fixed major organs (liver, kidney, spleen, lungs, and heart) and were stained with hematoxylin–eosin (HE) stain on a glass surface for a histopathologic study (LSI Medience Corp., Japan).

### Pharmacokinetic experiments

The 125-Iodinated HemoAct was prepared using our previously reported procedures^34^. The sample was diluted by non-labeled HemoAct to adjust the protein concentration before use. Wistar rats (269 ± 5 g, male) were anesthetized with diethylether. The HemoAct solution was injected intravenously via a tail vein (40 mg/kg, 0.2 mL/100 g, 1.5 × 10^6^ cpm/rat) (*n* = 6). At the time-points of 3, 10, 30 min, and 1, 3, 6, 12, and 24 h after the infusion, a heparinized syringe was used to collect 200 μL of blood from the lateral tail vein. A centrifuge (3000 rpm, 10 min) was used for removal of the blood cell components. Subsequently, the levels of ^125^I in the plasma were ascertained by measuring their radioactivity using an automatic gamma counter (2480 WIZARD2; PerkinElmer Inc.). Acid precipitability of the recovered radionuclide was confirmed using trichloroacetic acid. The rats were sacrificed by hemorrhage at the end of the experiments. Their vital organs (liver, kidney, spleen, lung, and heart) were isolated carefully. The weight and radioactivity of the excised organs were measured. As reference groups, the ^125^I-Hb and ^125^I-HSA solutions were administered similarly into rats [^125^I-Hb group (270 ± 9 g, 10 mg/kg, *n* = 6) and ^125^I-HSA group (260 ± 11 g, 30 mg/kg, *n* = 6)].

### Data analysis

Pharmacokinetic analyses were conducted after the administration of ^125^I-HemoAct using a non-compartment model. Pharmacokinetic parameters were calculated using the moment analysis program available with Microsoft Excel, as reported previously^35^. Data are shown as the mean ± SD for the indicated number of animals. Significant differences between groups were inferred using two-tailed unpaired Student’s *t*-tests. Probability values of *p* < 0.05 were inferred as statistically significant.

## Additional Information

**How to cite this article**: Haruki, R. *et al.* Safety Evaluation of Hemoglobin-Albumin Cluster “HemoAct” as a Red Blood Cell Substitute. *Sci. Rep.* **5**, 12778; doi: 10.1038/srep12778 (2015).

## Supplementary Material

Supplementary Information

## Figures and Tables

**Figure 1:**
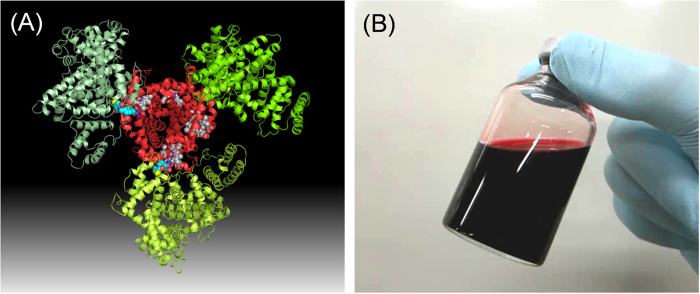
HemoAct. (**A**) Molecular structure of HemoAct in which an Hb core is wrapped covalently by three HSAs^19^. (**B**) HemoAct solution (20 g/dL) in PBS (pH 7.4).

**Table 1:** Pharmacokinetic parameters of ^125^I-HemoAct, ^125^I-HSA, and ^125^I-Hb after intravenous administration to rats (*n *= 6). T_1/2_, half-life; MRT, mean residence time; CL_total_, clearance; *V*_dss_, volume of distribution at steady state; AUC, area under the concentration-time curve. Each value represents the mean ± SD (*n* = 6). **p < 0.01 vs. ^125^I-HSA.

	T1/2 (h)	MRT (h)	CLtot (mL/hr)	Vdss (mL)	AUC (% of dose/ mL h)	
^125^I-Hb	0.53 ± 0.1	0.74 ± 0.14	16.5 ± 2.4	12.0 ± 1.3	6.2 ± 0.9	
^125^I-HSA	11.0 ± 0.67	15.0 ± 0.88	0.95 ± 0.07	14.3 ± 1.4	105.5 ± 8.2	
^125^I-HemoAct	18.5 ± 1.7**	26.3 ± 2.5**	0.50 ± 0.07**	13.0 ± 0.74	203.4 ± 23.2**	
