# Supplementary material for: Safety Evaluation of Hemoglobin-Albumin Cluster “HemoAct” as a Red Blood Cell Substitute
Source: Sci Rep. 2015 Jul 29;5:12778. doi: 10.1038/srep12778 (PMC4518235; doi:10.1038/srep12778)
Supplement: Supplementary Information [file srep12778-s1.doc]

=Supplementary Information=

**Safety Evaluation of Hemoglobin-Albumin Cluster “HemoAct” as a Red Blood Cell Substitute**

Risa Haruki^1^, Takuya Kimura^1^, Hitomi Iwasaki^1^, Kana Yamada^1^, Ikuo Kamiyama^2^, Mitsutomo Kohno^2^, Kazuaki Taguchi^3^, Saori Nagao^4^, Toru Maruyama^4^, Masaki Otagiri^3^ & Teruyuki Komatsu^1^

^1^Department of Applied Chemistry, Faculty of Science and Engineering, Chuo University, 1-13-27 Kasuga, Bunkyo-ku, Tokyo 112-8551, Japan; ^2^Department of Thoracic Surgery, School of Medicine, Keio University, 35 Shinanomachi, Shinjuku-ku, Tokyo 160-8582, Japan; ^3^Faculty of Pharmaceutical Sciences, Sojo University, 4-22-1 Ikeda, Nishi-ku, Kumamoto 860-0082, Japan; ^4^Department of Biopharmaceutics, Graduate School of Pharmaceutical Sciences, Kumamoto University, 5-1 Oe-Honmachi, Chuo-ku, Kumamoto 862-0973, Japan.

Correspondence and request for materials should be addressed to T.Ko. (komatsu@kc.chuo-u.ac.jp)

**Methods**

**Synthesis of XLHb.** Freshly prepared DMSO solution 1,6-bismaleimide hexane (BMH) (6 mM, 3.0 mL) was added dropwise to the pure carbonyl bovine Hb solution (0.1 mM, 30 mL in PBS) in a round-bottom flask (100-mL volume). After stirring for 5 h in CO atmosphere under dark conditions at 4 °C, the reactant was loaded onto a gel filtration chromatography (GFC) with a Sephadex G25 (superfine) column to remove unreacted crosslinker. The eluent of Cys93(β)-Cys93(β) crosslinked Hb (XLHb) was concentrated to [Hb] of 5 g/dL (Vivaspin 20 ultrafilter, 10 kDa MWCO; Sartorius AG). Native–PAGE exhibited one band at the same position of Hb. In contrast, SDS PAGE demonstrated two bands at 16 kDa and 32 kDa. The later indicated the formation of ββ-crosslinked Hb. The cysteinyl thiol assay of XLHb using 4,4’-dithiopyridine showed that both thiol groups of Cys-93(β) of Hb were completely bridged with BMH.

**Results**

**Figure S1** PT and APTT values of blood/(protein (HemoAct or HSA)) mixture suspension ([protein] = 0, 10, 20, and 40 vol%). Each bar represents the mean ± SD (*n* = 4).

**Figure S2** Time courses of body weights of rats after injection of HemoAct and HSA. Each data point represents the mean ± SD (*n* = 3).

**Figure S3** Weight of major organs recovered from rats (% of body weight, BW) after injection of HemoAct and HSA. Each bar represents the mean ± SD (*n* = 3).

| **Table S1** Serum biochemical assays | | | |
| --- | --- | --- | --- |
|  | Control | HSA | HemoAct |
| Total protein (g/dL) | 5.4 ± 0.0 | 5.4 ± 0.2 | 5.3 ± 0.4 |
| Albumin (g/dL) | 2.1 ± 0.0 | 2.2 ± 0.1 | 2.1 ± 0.2 |
| Albumin/globlin ratio (-) | 0.66 ± 0.03 | 0.70 ± 0.0 | 0.66 ± 0.04 |
| AST (U/L) | 82 ± 22 | 80 ± 27 | 68 ± 21 |
| ALT (U/L) | 28.3 ± 0.9 | 26.7 ± 2.1 | 29.7 ± 1.7 |
| γ-GTP (U/L) | 0.3 ± 0.5 | 0.3 ± 0.5 | 0.0 ± 0.0 |
| Total bilirubin (mg/dL) | 0.0 ± 0.0 | 0.1 ± 0.1 | 0.1 ± 0.0 |
| Direct bilirubin (mg/dL) | 0.0 ± 0.0 | 0.0 ± 0.0 | 0.0 ± 0.0 |
| Creatinine (mg/dL) | 0.23 ± 0.02 | 0.19 ± 0.04 | 0.21 ± 0.06 |
| Urea nitrogen (mg/dL) | 20.6 ± 1.2 | 18.4 ± 1.9 | 17.5 ± 2.0 |
| Uric acid (mg/dL) | 1.0 ± 0.2 | 1.0 ± 0.3 | 1.0 ± 0.3 |
| Amylase (U/L) | 2563 ± 190 | 2596 ± 105 | 2414 ± 203 |
| Total cholesterol (mg/dL) | 66 ± 5 | 66 ± 4 | 52 ± 5 |
| Free cholesterol (mg/dL) | 14 ± 4 | 16 ± 2 | 15 ± 2 |
| β-Lipoprotein (mg/dL) | 119 ± 61 | 113 ± 44 | 104 ± 20 |
| HDL cholesterol (mg/dL) | 27 ± 5 | 28 ± 2 | 20 ± 3 |
| Triglyceride (mg/dL) | 72 ± 38 | 73 ± 38 | 53 ± 15 |
| Total lipid (mg/dL) | 288 ± 34 | 294 ± 58 | 234 ± 14 |
| Free fatty acid (mEq/I) | 0.66 ± 0.18 | 0.57 ± 0.09 | 0.40 ± 0.16 |
| Phospholipid (mg/dL) | 116 ± 4 | 122 ± 16 | 101 ± 7 |
| K (mEq/I) | 4.0 ± 0.3 | 4.2 ± 0.7 | 4.2 ± 0.4 |
| Ca (mg/dL) | 10.3 ± 0.3 | 10.1 ± 0.3 | 10.1 ± 0.1 |
| Inorganic P (mg/dL) | 9.3 ± 0.7 | 9.5 ± 1.3 | 8.7 ± 1.0 |
| Mg (mg/dL) | 2.3 ± 0.0 | 2.3 ± 0.1 | 2.2 ± 0.2 |
| Fe (mg/dL) | 225 ± 32 | 213 ± 36 | 220 ± 7 |
| Cu(μg/dL) | 111 ± 5 | 104 ± 5 | 106 ± 8 |
| Each value represents the mean ± SD (*n* = 3). | | | |


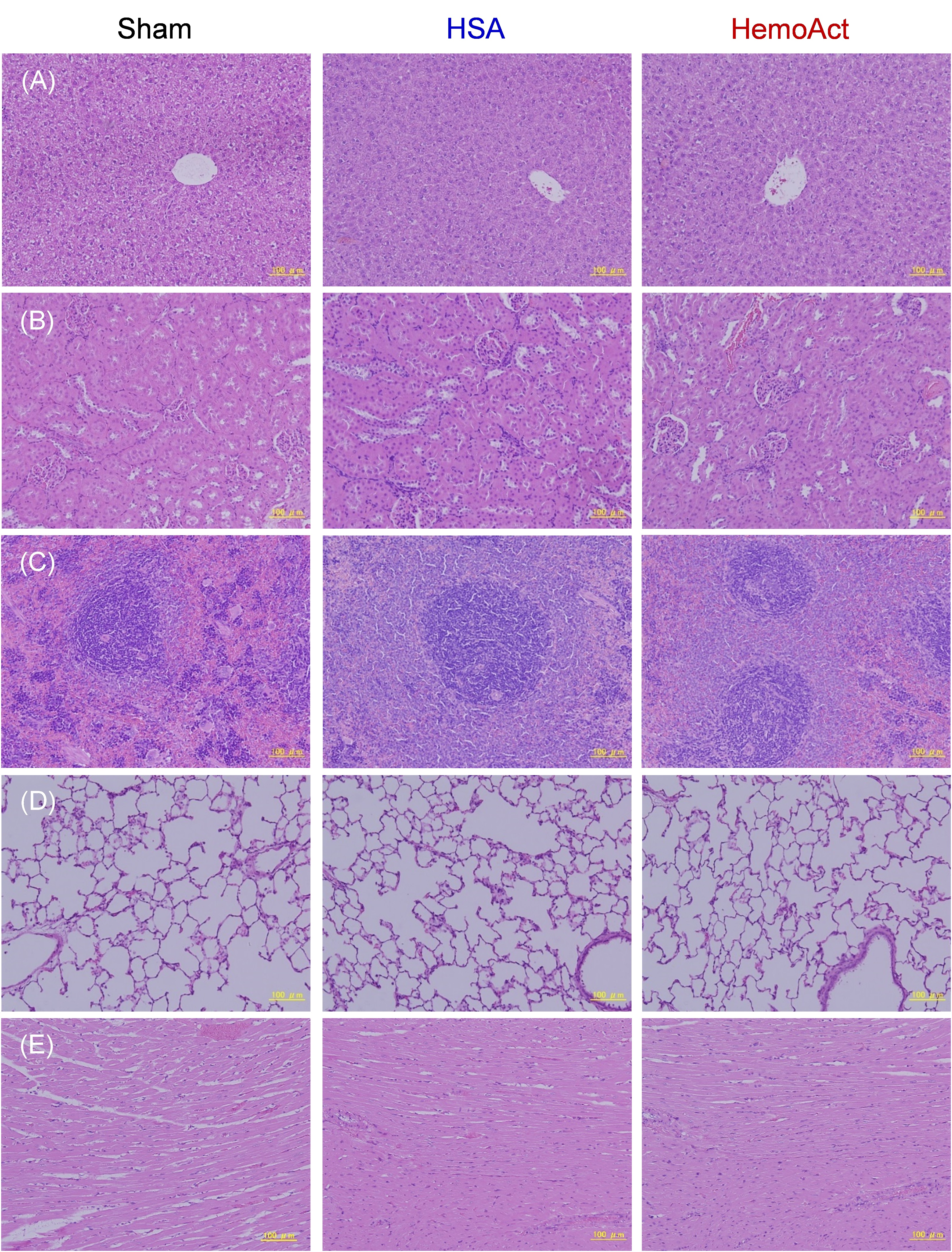


**Figure S4** Microscopic observations of stained specimens of vital organs recovered from rats after infusions of HemoAct and HSA. Hematoxylin‒eosin stain: (A) liver, (B) kidney, (C) spleen, (D) lung, and (E) heart.
